# Degradation of insecticides used for indoor spraying in malaria control and possible solutions

**DOI:** 10.1186/1475-2875-10-307

**Published:** 2011-10-18

**Authors:** Mthokozisi M Sibanda, Walter W Focke, Frederick JWJ Labuschagne, Lumbidzani Moyo, Nontete S Nhlapo, Arjun Maity, Herminio Muiambo, Pedro Massinga, Nico AS Crowther, Maureen Coetzee, Gordon WA Brindley

**Affiliations:** 1 Institute of Applied Materials, Departments of Chemistry and Chemical Engineering, University of Pretoria, Lynwood Road, Pretoria 0002, South Africa; 2 Polymers and Composites, MSM - CSIR, 1 Meiring Naude Road, Brummeria, Pretoria 0001, South Africa; 3 Department of Chemistry, Eduardo Mondlane University, P.O. Box 257, Maputo, Mozambique; 4 Department of Statistics, University of Pretoria, Lynwood Road, Pretoria 0002, South Africa; 5 Malaria Entomology Research Unit, School of Pathology, Faculty of Health Sciences, University of the Witwatersrand and the National Institute for Communicable Diseases, 1 Modderfontein Road, Sandringham 2131, South Africa

**Keywords:** Indoor residual spray, DDT, pyrethroid, carbamate, stabilization

## Abstract

**Background:**

The insecticide dichloro-diphenyl-trichloroethane (DDT) is widely used in indoor residual spraying (IRS) for malaria control owing to its longer residual efficacy in the field compared to other World Health Organization (WHO) alternatives. Suitable stabilization to render these alternative insecticides longer lasting could provide a less controversial and more acceptable and effective alternative insecticide formulations than DDT.

**Methods:**

This study sought to investigate the reasons behind the often reported longer lasting behaviour of DDT by exposing all the WHO approved insecticides to high temperature, high humidity and ultra-violet light. Interactions between the insecticides and some mineral powders in the presence of an aqueous medium were also tested. Simple insecticidal paints were made using slurries of these mineral powders whilst some insecticides were dispersed into a conventional acrylic paint binder. These formulations were then spray painted on neat and manure coated mud plaques, representative of the material typically used in rural mud houses, at twice the upper limit of the WHO recommended dosage range. DDT was applied directly onto mud plaques at four times the WHO recommended concentration and on manure plaques at twice WHO recommended concentration. All plaques were subjected to accelerated ageing conditions of 40°C and a relative humidity of 90%.

**Results:**

The pyrethroids insecticides outperformed the carbamates and DDT in the accelerated ageing tests. Thus UV exposure, high temperature oxidation and high humidity *per se *were ruled out as the main causes of failure of the alternative insecticides. Gas chromatography (GC) spectrograms showed that phosphogypsum stabilised the insecticides the most against alkaline degradation (i.e., hydrolysis). Bioassay testing showed that the period of efficacy of some of these formulations was comparable to that of DDT when sprayed on mud surfaces or cattle manure coated surfaces.

**Conclusions:**

Bioassay experiments indicated that incorporating insecticides into a conventional paint binder or adsorbing them onto phosphogypsum can provide for extended effective life spans that compare favourably with DDT's performance under accelerated ageing conditions. Best results were obtained with propoxur in standard acrylic emulsion paint. Similarly, insecticides adsorbed on phosphogypsum and sprayed on cattle manure coated surfaces provided superior lifespans compared with DDT sprayed directly on a similar surface.

## Background

The World Health Organization (WHO) Global Malaria Action Plan promotes indoor residual spraying (IRS) as a primary operational vector control intervention to reduce and ultimately eliminate malaria transmission. In some southern African countries DDT is regarded as the most effective insecticide for this purpose. Depending on the dosage and substrate nature, DDT retains its efficacy against malaria vectors for up to 12 months. In South Africa DDT was temporarily replaced with the pyrethroid deltamethrin between 1996 and 1999. However, DDT was reintroduced in 2000 when malaria transmission reached epidemic proportions. The failure of the pyrethroid was attributed to the return of the major vector mosquito, *Anopheles funestus *that was shown to be resistant to pyrethroids but fully susceptible to DDT [1,2]. Other WHO-approved pyrethroid, organophosphate and carbamate insecticides are limited in effective IRS residual life. Furthermore, repeated application of these alternatives is required in order to provide year-round protection and this significantly increases the costs of IRS [3]. Formulations based on micro-encapsulated insecticides have been tested with great success [4-7]. These results show that shielding the insecticides from the outside environment stabilizes them against premature degradation. However, the higher costs associated with such formulations may limit their widespread implementation as replacements for DDT in IRS.

The stability of WHO-approved insecticides for IRS is affected by the pH of the environment [8-14], temperature [15-19], exposure to ultraviolet (UV) light [20-30] and the availability of degrading bacteria [31-36]. Pyrethroids, organophosphates and carbamates degrade via hydrophilic attack of the carboxylic and carbamic ester linkages [11-13]. DDT undergoes alkaline dechlorination to yield DDE [14].

On thermal exposure, phenyl carbamates representative of bendiocarb and propoxur degrade to the corresponding phenol and methylisocyanate [15]. Pyrethroids transform by isomerization, ester cleavage and primary oxidation of the final products [16,17]. Organophosphates, e.g. malathion, initially isomerize to S-alkyl organophosphates before they eventually decompose [18]. The primary step in thermal decomposition of *p,p'*-DDT is the elimination of HCl, resulting in the formation of *p,p'*-DDE at 152°C [19]. DDE starts to volatilize at the onset of the process. The decomposition temperature is dependent on the type of DDT, for *o,p'*-DDT the decomposition starts at higher temperatures.

All the insecticides classes, except for pyrethroids to some extent, are degraded by exposure to ultraviolet light. Fenitrothion undergoes photo-oxidation on the benzene methyl group to form a carboxylic acid group. It may also undergo oxidation on the ─P═S moiety group to form the oxon and ester cleavage to form the corresponding phenol [20]. Malathion is generally stable to photolysis. This may be due to a lack of chromophores to absorb radiation in the UV- range [21]. Pirimiphos-methyl photodegrades rapidly forming 2-diethylamino-6-methylpyrimidin-4-ol as the major degradation product [22]. Carbamate photodegradation involves the cleavage of the carbamic acid ester to form the corresponding alkyl phenyl ether or alkyl phenol ether [23]. Carbamates isomerize on irradiation through the photo-Fries re-arrangement. Photo decomposition of carbamates also produces cholinesterase inhibitors [24]. DDT undergoes homolytic cleavage of the C─Cl bond on the trichloromethyl group to form DDD as the product [25]. DDE may be produced due to exothermic effects of the initial photodechlorination stage. The overall photodechlorination is proposed to be dominated by the sequential dechlorination pathway with each successive dehalogenation proceeding more slowly. Apart from etofenprox, all pyrethroids are structurally similar. They all feature the characteristic carboxylic ester moiety, the dihalogen substituted vinyl moiety and the phenoxy ether group. Etofenprox only contains the phenoxy ether group. The carboxylic ester moiety is susceptible to photodegradation and hence decarboxylation of these insecticides is the main route of photodegradation [26,27]. However photodecarboxylation of these pyrethroids is a minor transformation process accounting for not more than 15% of the transformation products [28]. The amount of decarboxylation depends on the halogen substituents on the vinyl moiety, i.e. the more electronegative the halogen the more the decarboxylation. The main process of transformation of these pyrethroids is photo-isomerization (cis/trans and E/Z) on the triplet diradicals formed on exposure to ultraviolet light (>300 nm), e.g. deltamethrin [29], cypermethrin and cyhalothrin [30]. Cyfluthrin is subject to photolysis rapidly forming 4-fluorophenoxybenzaldehyde via ester hydrolysis and the release of cyanide ion from the corresponding cyanohydrin [21].

Extensive work has been done on the fundamentals of insecticide biodegradation to develop bioremediation techniques to detoxify contaminated environments [31]. Microbiological degradation of insecticides relies on the availability of organisms that can secrete specific degrading enzymes. Bacteria and fungi have the ability to produce these enzymes under both aerobic and anaerobic conditions. Carbamates, pyrethroids and organophosphates have a common ester moiety that provides a route for bacterial-mediated enzymatic biotransformation. The enzyme groups that are able to hydrolyse these ester moieties include carboxyesterases for carbamic and carboxylic esters, and phosphotriesterases and carboxylesterases for triphosphate esters [32]. DDT is degraded by oxygenases and dehydrogenase enzymes [33]. A period of adaptation is required before degrading microbes manage to establish themselves and significant degradation of the insecticide is observed [34]. This acclimation period entails the building of viable microbial colonies that have a capacity to induce enhanced degradation [35]. The length of the acclimation period is also affected by the availability of suitable metabolic substrates for growth. These can be a carbon source, a mineral nutrient source, or both. The presence of alternative carbon substrates and nutrients generally increases the rate of biodegradation. This is supported by the observed increase in the rate of DDT degradation in highly fertilized soils [36].

Mineral powders such as bentonite, gypsum, montmorillonite, kaolin, attapulgite, diatomite etc. have been used as insecticide carriers in granular insecticide formulations [37,38]. These minerals have the ability to slowly release the adsorbed insecticide into the environment [39,40]. Lagaly [41] presented an excellent review on pesticide-clay interactions and formulations relevant to formulating insecticide laden clay formulations.

Impurities can mediate catalytic degradation of insecticides adsorbed on soil surfaces. Stability can be improved by deactivating surface active sites responsible [42]. Additives such as polyethylene glycol are reportedly very effective deactivators. A useful property of mineral powder carriers is that they can stabilize photolabile and thermolabile insecticides [43-45].

Our ultimate aim is to develop long-lasting, "green" and cost effective alternatives to DDT for IRS. This paper reports on investigations directed towards finding ways to extend the active life of insecticides formulated in environmentally stable, pesticide-based "whitewash" or equivalent "paint". The goals of this investigation were to overcome the time-limited effectiveness of current WHO-approved DDT alternatives by evaluating the interactions between selected insecticides with the micro-environment on which they are applied and also with low-cost paint ingredients.

## Methods

### Materials

The technical grade insecticides used in this study are listed in Table 1 together with their suppliers. Phosphogypsum is a waste product generated in the production of phosphoric acid. It was chosen because it is readily available in immense quantities and because the phosphoric acid impurity imparts an acidic nature to this filler. Phosphogypsum powder was supplied by Gypsum Industries. The d (0.5) particle size was 7.6 μm (Mastersizer Hydrosizer 2000). Attapulgite with a d (0.5) of 32.0 μm was supplied by G&W Base Minerals. The particles of this mineral have an elongated shape with parallel channels through the lattice that impart a high adsorption capacity and a high surface area [38]. Calcium bentonite powder (d_50 _= 20.1 μm) was supplied by G&W Base Minerals. Dellite 67G organoclay, a montmorillonite intercalated with ditallowdimethylammonium (d_50 _= 12.2 μm) was supplied by Laviosa.

**Table 1:** Insecticides tested and WHO recommended dosage range for IRS

Insecticide class/Insecticide	**IRS dosage g/m**^**2**^	Activity* months	Supplier
**Organochlorine**			
DDT	1 - 2	>6	Avima
**Carbamates**			
Bendiocarb	0.1 - 0.4	2-6	Bayer
Propoxur	1 - 2	3-6	Avima
**Pyrethroid**			
Alphacypermethrin	0.02 - 0.03	0.02-0.03	Bilag

*Duration of effective action

A sulphonated mimosa extract (Mimosa WS supplied by Mimosa Extract Company) was used as dispersant. Makeean Polymers MCP 503 acrylic copolymer emulsion was used as binder. The emulsion had a solids content of 56 wt.% and a pH of 7.5 - 8.5.

Ethanol (96% Univar rectified), methanol (Univar) and acetone (Univar) were supplied by Merck. Two soil samples were used to prepare substrate surfaces. They were collected at Thomo (Clay 10%, Silt 25.0% and coarse sand 59.8%) and Makoya (clay 10%, silt 10% and coarse sand 95.1%) locations in Mozambique. The pH values of the soil samples were 7.1 and 6.5 respectively.

#### Accelerated ageing mediated by temperature, ultra-violet light or high humidity

Polytetrafluoroethylene (PTFE) infrared sample cards supplied by Life and Analytical Sciences (PTY) Ltd were used as sample holders. PTFE was chosen as substrate on account of its chemical inertness, UV and temperature stability as well as its transparency in the infrared range 1300 cm^-1 ^to 3400 cm^-1 ^wave numbers. Neat insecticide films were deposited on the substrate by coating it with a 1 wt.% solution of the active in acetone and allowing the solvent to evaporate.

The state of the insecticides deposited on the Teflon substrates was followed as a function of ageing time using Fourier Transform Infrared (FTIR) spectroscopy. A Perkin Elmer Spectrum 100 spectrometer was used in transmission mode. The resolution was 1 cm^-1 ^and 20 scans were recorded per sample.

A Labcon economy type EFDO oven, set at 80°C, was used for high temperature ageing. Samples were exposed to this elevated temperature for consecutive 50 h periods. FTIR spectra were recorded after each exposure period. A Labcon model FSIM forced circulation incubator was used for humidity ageing. The incubator controlled the temperature to within ±0.2°C and humidity to within ± 2% ℜH. The samples were exposed to a relative humidity of 90% at 60°C for 168 h. Accelerated artificial weathering was conducted in a QUV tester fitted with A340 UV lamps. A dry cycle was used with the temperature set at 63°C and an irradiance of 0.67 W.m^-2^.

### Hydrolytic stability of the insecticides

Since most insecticides are effectively insoluble in water, the following procedure was used to explore the effect of mineral powders on hydrolytic stability. Stock solutions containing ca. 1% insecticide were prepared by first dissolving 8 g bendiocarb or alphacypermethrin in 800 g acetone and then adding 32 g of distilled water. Mineral powder (1 g) was added to two sets of six Schott glass bottles. To this was added 21 g of the respective insecticide stock solutions. The top of the bottles were sealed with Parafilm before capping and then wrapped in aluminium foil to protect against light exposure. The bottles were agitated on a shaker table at frequency of 180 cycles per minute during the ageing period at ambient conditions (temperature ranging from ca. 15°C to 25°C). Two sets of solutions (without mineral powders) for each insecticide were used as reference and control standards respectively. The Reference sample was stored in a fridge and the (negative) Control sample was kept under the same conditions as the test samples. A small amount of sodium ethoxide, a strong alkali, was added to another pair of Schott glass bottles containing the bendiocarb and alphacypermethrin stock solutions. These samples provided a positive control and were prepared in order to confirm the instability of both insecticides in highly alkaline environments.

The degradation of the insecticide in the presence or absence of mineral powders was followed using a Varian Star model 3400CX gas chromatograph fitted with an Agilent HP 5 GC column. The column length was 30 m and the internal diameter 0.25 mm with a film thickness of 0.25 μm. The carrier gas was helium and the gas split ratio was 1:30. A flame ionization detector was used. For bendiocarb the initial oven temperature was 120°C, a holding time of 1 minute, a ramp rate of 25°C.min^-1^, final temperature of 250°C and a final holding time of 10 min. The injector temperature was 200°C while the detector temperature was set at 265°C. For alphacypermethrin the initial oven temperature was set at 245°C and the temperature was immediately ramped at a rate of 25°C.min^-1 ^to the final temperature of 275°C where it was held for 10 minutes. The injector and detector temperatures were both set at 250°C.

#### Spray surfaces and insecticide formulations for ageing and bio-assaying

Soil samples were mixed with warm water to form a thick paste. This paste was then packed in substrate holders made from high density polyethylene (HDPE). These were round shallow dishes with diameter 11.6 cm and depth 2 cm or diameter 15.5 cm and depth 2.5 cm. The mud substrates were then oven dried at 90°C for 24 h. One set of the dried soil plaques was coated with fresh cattle manure diluted with some water. These represented the microbiologically active surfaces typically found in rural mud houses.

### Preparation of spray "paints"

Paints were prepared using alphacypermethrin (pyrethroid), bendiocarb and propoxur (carbamates) and DDT (organochlorine). Organophosphates were excluded because they were considered too unstable. Insecticides were dissolved in just enough acetone to completely dissolve the insecticides. Since the insecticides are all water insoluble, the insecticides precipitated on the gypsum when the solution was dispersed into the phosphogypsum slurry. Next, dispersant was added at a loading of 2 wt.% based on the mass of gypsum used. This level was chosen on the basis of its viscosity reducing effect on a 50 wt.% phosphogypsum slurry. These dispersion formulations were sprayed on mud plaques. Experiments conducted at a later stage established that more stable dispersions were obtained by a slight modification of the procedure. In this method the insecticide was dissolved in just sufficient acetone to allow complete wetting of the dry phosphogypsum powder. This mixture was then slurried into water using vigorous stirring and then adding the same amount of dispersant. This second set of dispersion formulations was sprayed onto the cattle manure-coated mud plaques.

The following three types of "paints" formulations were prepared and tested:

• **Gypsum "white wash"**. Insecticide-coated phosphogypsum slurry (50 wt.% solids with 1 wt.% dispersant) was used as is. The final insecticide content based on dry solids was 0.37 wt.% alphacypermethrin and 9 wt.% bendiocarb. The dispersions were sprayed on soil surfaces and cattle manure coated surfaces.

• **Gypsum "white wash" with minimal acrylic binder**. Phosphogypsum (45 wt.%), paint binder (4.5 wt.%), dispersant (0.9 wt.%), water (45 wt.%) plus bendiocarb (4.5 wt.%) was used. The final insecticide content based on dry solids was 8 wt.%. This "paint" was sprayed on the mud surfaces. The idea behind adding the acrylic binder was to improve adhesion of the insecticide to the house wall surface.

• **Acrylic paint**. Carbamate insecticides (bendiocarb or propoxur), dissolved in a minimal amount of acetone, were mixed into the neat acrylic emulsion. The final insecticide content was 15.2 wt.% based on dry solids. This "paint" was sprayed on standard soil surfaces.

### Surface spraying

The insecticide "paints" were deposited on the substrates using an Aircraft Pneumatic Systems, Model SG AS 1001A spray gun. The feed to the spray gun was by gravity. The nozzle diameter was 1.4 mm and the working pressure 2 bar. The spray pattern was adjusted such that the widest possible spray width was achieved. A smaller Aircraft Pneumatic Systems Model SG A138 spray gun was used to spray the DDT acetone solutions. The feed to the spray gun was via suction. This nozzle diameter was 0.8 mm and a working pressure 3.45 bar. The compressed air used for spraying was tapped from a 6 bar compressed air line via a common manifold.

Each "paint" was sprayed onto soil plaques and cattle manure coated surfaces. Triplicate samples of each type was produced and tested. Since the present purpose was to study degradation under accelerated laboratory conditions, the final insecticide loading was set at twice the upper limit of the WHO recommended dosages [46] (Table 1). DDT was sprayed directly onto the standard soil surface and cattle manure coated surface as a 10 wt.% solution in acetone. Four times the recommended amount of DDT was employed on the soil substrates because it performed poorly when tested at twice the upper limit.

### Bioassays

The treated surfaces were subjected to accelerated ageing in a humidity cabinet set at 90% relative humidity and a temperature of 40°C. WHO bioassays methods [47] were used to periodically track the residual efficacy against mosquitoes. In these tests, 25 non-blood-fed female *Anopheles arabiensis *mosquitoes (KGB colony housed at the National Institute for Communicable Diseases, Johannesburg), 3-5 days old, were exposed for 30 minutes to samples of the treated or untreated substrate surfaces. Mortality rates were recorded 24 hours after initial exposure of the triplicates for each treated surface.

## Results

The results obtained for thermal ageing, UV degradation and resistance to high humidity are summarized in Table 2. As a group, the pyrethroids showed the greatest resistance to degradation whether mediated by elevated temperature, UV light exposure or high humidity. Selected members of this class survived more than 250 h oven ageing at 80°C, up to 150 h 0.67 W.m^-2 ^QUV exposure at 63°C and all were still present after 168 h high humidity ageing at 40°C and 90% ℜH. In contrast, DDT and the other classes were either completely degraded or lost after only 50 h of exposure to high heat or UV light.

**Table 2:** FTIR recorded performance under accelerated ageing conditions

Insecticide class	Insecticide	**Heat ageing**^**1**^	**UV degradation**^**2**^	**Humidity**^**3**^
**Organochlorine**	DDT	<50 h	<100 h	lost

**Organophosphate**	malathion	<50 h	<50 h	-
	fenitrothion	<50 h	<50 h	-
	pirimiphos-methyl	<50 h	<50 h	lost

**Carbamates**	bendiocarb	<50 h	<50 h	lost
	propoxur	<50 h	<50 h	lost

**Pyrethroids**	alphacypermethrin	>250 h	>250 h	present
	bifenthrin	<50 h	<150 h	present
	cyfluthrin	>250 h	<150 h	present
	deltamethrin	>250 h	<150 h	present
	lambdacyhalothrin	<150 h	<150 h	present

**Other**	etofenprox	<200 h	<150 h	present

^1 ^Oven ageing at 80°C in 50 h intervals; ^2^Dry cycle UV degradation in a QUV at 63°C and 0.67 W/m^2 ^in 50 h intervals; ^3 ^168 h Humidity ageing at 40°C and 90% ℜH

### Thermal ageing

**Figure 1:**
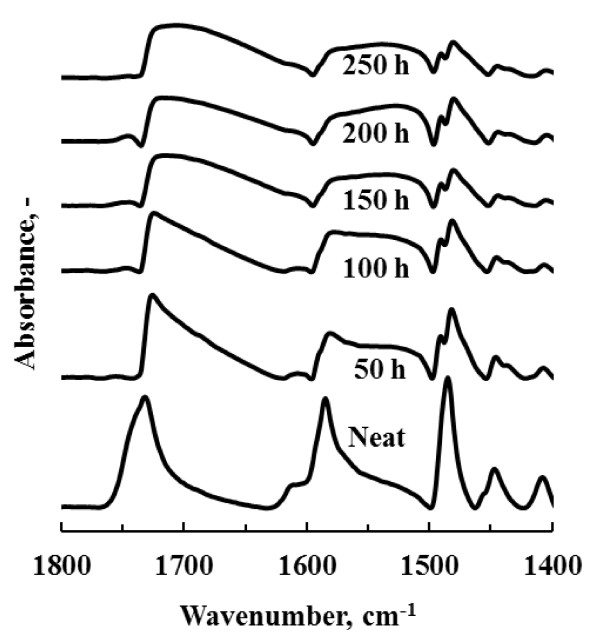
**FTIR spectra of alphacypermethrin deposited on a Teflon substrate as a function of oven ageing time at 80°C**.

The FTIR spectra (Figure 1) showed that alphacypermethrin was still present after 250 h exposure to a temperature of 80°C. However, a reduction and broadening in the characteristic absorption peaks is apparent after about 50 h suggesting isomerization of the parent compound and, possibly, partial volatilization. No DDT, organophosphate or carbamate was detectable by FTIR on the sample substrates after 50 h at this temperature.

### UV light exposure

**Figure 2:**
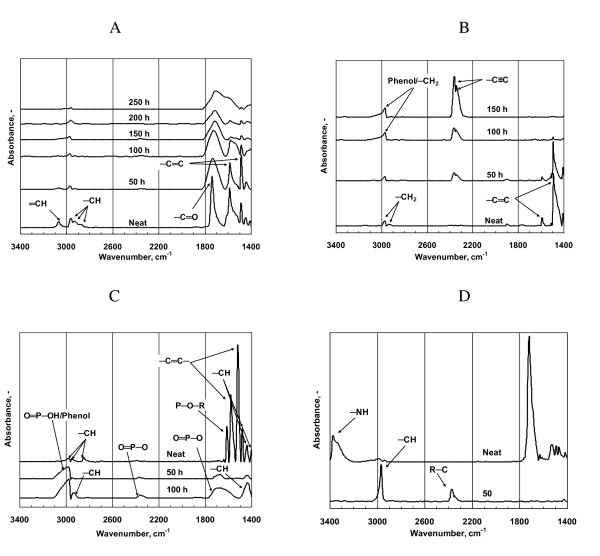
**FTIR spectra following QUV ageing: A. Alphacypermethrin; B. DDT; C. Fenitrothion; D. Bendiocarb**.

The temporal behaviour of the pyrethroid insecticides exposed to UV light is exemplified by the FTIR spectra recorded for alphacypermethrin (Figure 2A). The spectrum recorded for the neat insecticide shows strong and well-resolved absorption bands located at 1740 cm^-1 ^(ester carbonyl C=O stretch), 1605 cm^-1 ^and 1485 cm^-1 ^(aromatic C-C in-ring stretch modes). With increase in exposure time these initially very sharp bands broaden considerably and diminish in strength. However, they are still present following 250 h of UV light exposure. The observed broadening is attributed to the formation of degradation products with slightly shifted absorption bands that are observed superimposed on those of carbonyl and aromatic functional groups of the parent compound [48].

The temporal changes in the FTIR spectra for DDT are presented in Figure 2B. The strongest absorption band for DDT is the aromatic C-C in-ring stretch mode located at ca. 1500 cm^-1^. It is still present after 50 h of exposure but has completely disappeared after 100 h irradiation. The appearance of two new absorption bands 2355 and 2365 cm^-1 ^after merely 50 h UV light exposure indicate the onset of UV mediated degradation. This doubled dominated in the spectra recorded after 100 h and 150 h UV light exposure. The increased intensity of the absorption band at 2968 cm^-1 ^suggests that the aromatic rings were oxidized to phenols or quinones.

The absorption bands characteristic of fenitrothion disappeared within the first 50 h of ageing (Figure 2C). The spectrum of the degraded residue is consistent with the formation of phenols (ca. 2970 cm^-1^) and phosphates (ca. 2343 cm^-1^). Bendiocarb also degraded rapidly when exposed to UV light (Figure 2d). The strong absorption peak characteristic of the carbamate carbonyl has completely disappeared after 50 h of irradiation.

### Humidity ageing

**Figure 3:**
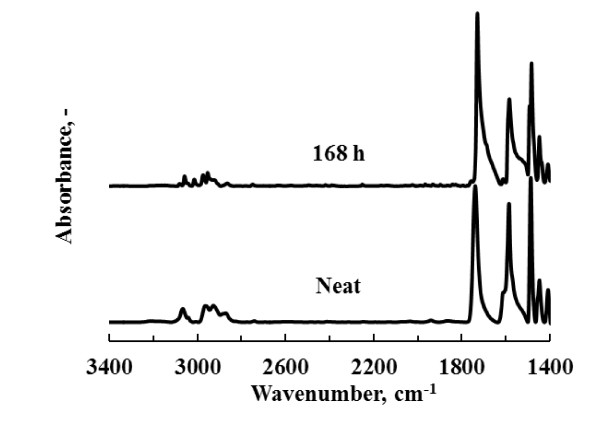
**FTIR spectra for neat alphacypermethrin and after ageing in a humidity chamber at 60°C and 90% ℜH for 168 h**.

The FTIR spectra for the samples that were exposed to a relative humidity of 90% at 60°C revealed that only the pyrethroids were still present after 168 h. The results for alphacypermethrin, given in Figure 3, are typical. DDT, pirimiphos-methyl and the carbamates were undetectable after this ageing period.

### Effect of mineral powder on insecticide hydrolysis rates

**Figure 4:**
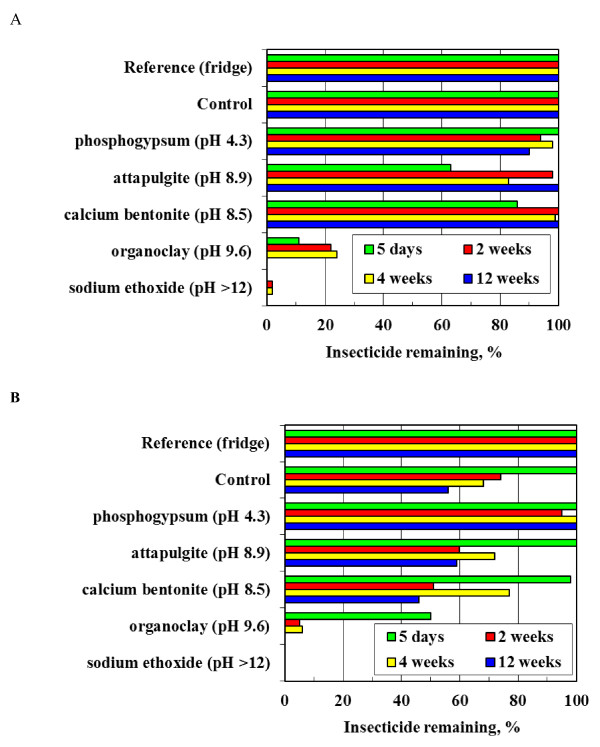
**Hydrolysis rates for: A) Bendiocarb and B) Alphacypermethrin on different mineral powders and suspended in acetone containing water**.

Figure 4 presents the effect of the mineral fillers on the persistence of bendiocarb (Figure 4A) and alphacypermethrin (Figure 4B) solutions in acetone containing 1 wt.% water. Adding catalytic amounts of sodium ethoxide caused, as expected, rapid hydrolysis of both insecticides. Bendiocarb was quite stable in this medium. No change in concentration was detected in this medium when aged at room temperature (Control sample). In contrast, the alphacypermethrin Control sample degraded over time. The concentration of both insecticides decreased rapidly in the presence of the organoclay filler. This does not necessarily mean that it was degraded in the presence of this substance. Co-intercalation of the insecticides into the clay galleries can also remove them from the liquid phase. Adding phosphogypsum appears to have improved the hydrolytic stability of alphacypermethrin whereas the opposite appears to be true for bendiocarb.

### Bioassays

**Figure 5:**
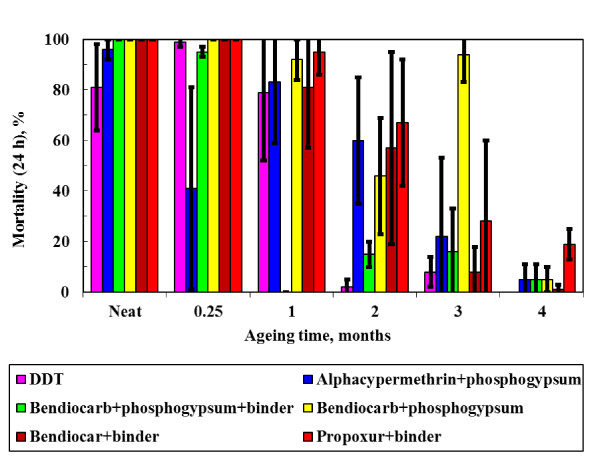
**Bioassay (Mortality after 24 h) results obtained on soil samples treated with insecticides and aged at 40°C and 90% ℜH for varying time periods**.

**Figure 6:**
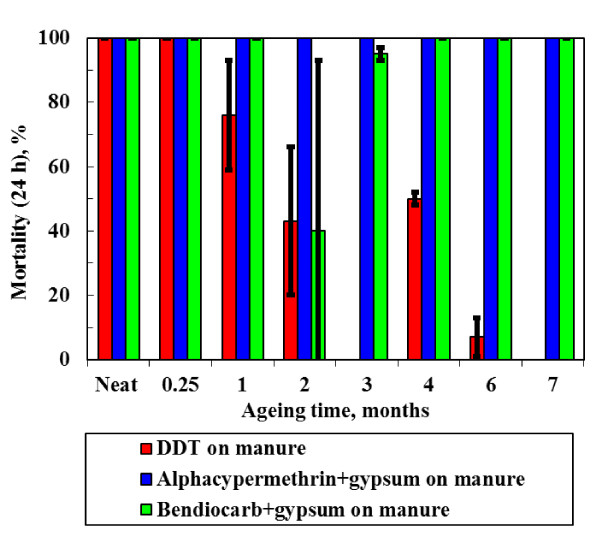
**Bioassay (24 h Mortality) results for manure coated soil surfaces treated with insecticides and aged at 40°C and 90% ℜH for varying time periods**.

Exploratory bioassay tests were performed using gypsum "white wash" with minimal acrylic binder option sprayed on mud and on dung coated mud surfaces. Propoxur performed well on both surfaces achieving 100% mortality after 24 h. However, alphacypermethrin failed the WHO criterion with an initial mortality of only 25%. This is attributed to a high solubility of this insecticide in the resin such that the surface concentration falls below the effective level. Furthermore, spray tests showed that the phosphogypsum adhered well to a variety of wall surfaces, i.e. addition of the acrylic emulsion was not necessary. These findings guided our selection of the formulations that were sprayed and performance-characterized by bioassay testing. The results for insecticidal "paints" applied to mud surfaces and cattle manure coated mud surfaces are presented in Figure 5 and Figure 6 respectively.

**Figure 7:**
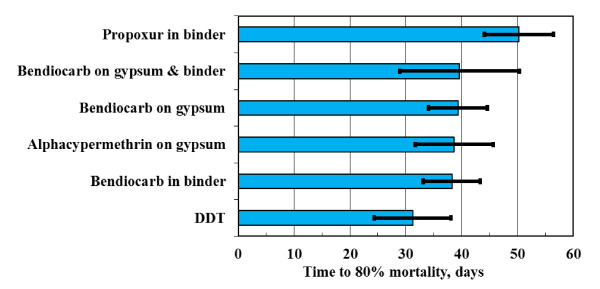
**WHO effective life time (> 80% Mortality after 24 h) for various treatments on mud surfaces. The error bars indicate the 95% confidence intervals determined via logistics regression**.

The WHO effectiveness criterion for IRS is a mosquito mortality exceeding 80% after 24 h following a 30-minute exposure. The data show considerable scatter but it is clear that most formulations fail by the second month of exposure to the accelerated laboratory ageing conditions. The data were fitted using logistic regression in order to allow statistically valid comparisons of the time to failure for each treatment. The times to reach 80% mortality were estimated from the fitted equations. The results are plotted in Figure 7 together with 95% confidence intervals. The following ranking (estimated time to failure in days indicated in brackets) was obtained for paints applied to mud surfaces:

DDT (31.3) < bendiocarb in binder (38.3) ≈ alphacypermethrin on gypsum (38.7) ≈ bendiocarb on gypsum (39.4) ≈ bendiocarb on gypsum with paint binder (39.6) < propoxur in acrylic paint binder (50.3)

However, the 95% confidence interval for DDT effective life time overlaps with that for all the other treatments except propoxur in the acrylic binder. This means that the difference in performance between DDT and the other paints on mud surfaces is not statistically significant. However, this outcome applies specifically to the procedure used for preparing the dispersion applied to the mud surfaces.

DDT showed similar performance on the dung coated mud surfaces with an estimated active time of 29.3 days and a 95% confidence interval of 25.3 days to 33.4 days. The overall compositions of the insecticide-on-gypsum slurries sprayed onto the mud and dung coated mud samples were identical. However, the improved preparation method was used in the latter case. Therefore it is not possible to make direct comparisons of the performance achieved on the dung surface surfaces with those found on the corresponding mud surfaces. However, mortality rates of 100% were achieved even after seven months of ageing for both the alphacypermethrin-on-gypsum and bendiocarb-on-gypsum sprayed onto manure-coated surfaces.

## Discussion

### Loss of insecticide

IRS relies on the fact that female mosquitos tend to settle on the walls inside dwellings where they may rest for a few days after they have taken a blood meal. The insecticides used in IRS are contact poisons that are applied to the inside walls of dwellings to kill the female mosquitoes in this resting phase. The activity of the insecticides deposited on such walls decreases over time. This may be due to volatilization or degradation processes, e.g. biodegradation, photodecomposition and alkaline hydrolysis. Volatilization occurs by evaporation for liquid insecticides and by sublimation for solid insecticides. The rate of vaporization increases with the ambient temperature and the degree of ventilation. In general, it can be estimated from equation (1) [49]:

(1) $$n_{A}\;\;=\;\;Sh\frac{D_{AB}P_{A}M_{A}}{zRT}$$

where *n*_A _is mass flux in kg.m^2^.s^-1^; *Sh *is the dimensionless Sherwood number; *D*_AB _is the diffusion coefficient in m^2^.s^-1^; *P*_A _is the vapour pressure of the insecticide in Pa; *z *is a characteristic length of the system measured in m the diffusion distance in m, taken here as half the width of the house; *R *is the gas constant 8.314 J.mol^-1^.K; *T *is the absolute temperature in K; and *M*_A _is the molar mass of the insecticide expressed in kg.mol^-1^.

**Figure 8:**
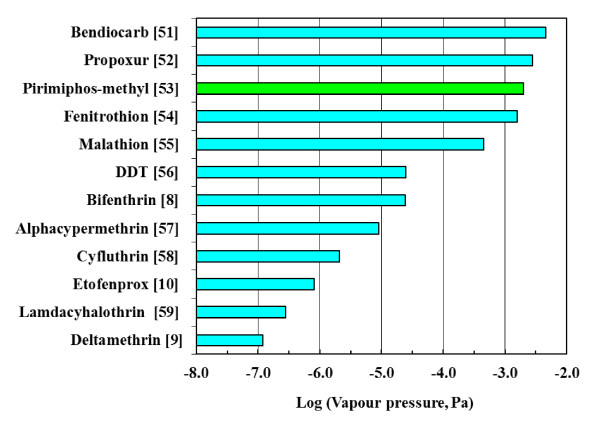
**Log vapour pressure plots of the insecticides at 25°C but for pirimiphos-methyl at 20°C. References are given in brackets after each insecticide name**.

The rate of loss is least in the absence of any air movement. In this scenario the rate of mass loss is determined by rate of diffusion of the insecticide through the stagnant air layer and the Sherwood number assumes the value of unity while *z *is the diffusion distance in meters normal to the wall surface. For preliminary estimation purpose it is taken as half the width of the house. The diffusion coefficients in air of the present insecticides were estimated using Fuller's method [50]. Published vapour pressure data at 25°C [8-10,51-59] are summarized in Figure 8. Assuming a diffusion distance of 2 m, ambient temperature of 25°C, using the WHO IRS dosage recommendations and applying equation (1) leads to the following estimates for the bioactive life in years:

Fenitrothion, 136; pirimiphos-methyl 110; bendiocarb, 11; propoxur 96; malathion 457; DDT, 7726; and alphacypermethrin 319.

Raising the ambient temperature has a dramatic effect. For example, at 40°C the persistence of fenitrothion is reduced to 24 years. Ventilation further increases the rate of volatilization. In order to get a rough estimate of the impact of this factor, a flat plate approximation model for the wall is assumed with the air flow parallel to the wall at a velocity *V *in m.s^-1^. For this geometry the characteristic length *z *corresponds to the length of the wall *L *and the Sherwood number is given by [60]:

(2) Sh  =  0.667ReSc1/3

where Re is the Reynolds number (*VL/v*); *Sc *is the Schmidt number (*v/D*_*AB*_); *v *is the kinematic viscosity in m^2^.s^-1^.

Typical velocities that could be encountered inside a house range from ca. 0.001 m.s^-1 ^to about 1 m.s^-1^. Using these values and typical property values for air at ambient conditions reveals that the active life times indicated above could be reduced by 4 to 100 times. This means that, with the possible exception of bendiocarb, the loss of activity of the WHO insecticides experienced in the field cannot be attributed to volatilization! The implication is that the loss of activity over time must be associated with degradation processes.

### Ageing processes

In general organic insecticides degrade when exposed to elevated temperatures (thermal degradation); sunlight (photodegradation); moisture (e.g. hydrolysis), and microbial attack. The laboratory tests used in this study simulated relatively extreme conditions of temperature, ultra violet light exposure, humidity, alkalinity and microbiologically activity. The temporal performance of neat and stabilized WHO-approved insecticides samples were compared to that of neat DDT. It was found that DDT alternatives stabilized through incorporation in an acrylic binder or precipitated on acidic filler outlasted DDT when applied to dung coated surface and performed at least at a similar level when applied to soil surfaces.

The loss of DDT and carbamate insecticide activity under the ageing condition of 80°C is attributed to a combination of thermal degradation and volatilization. The latter speculation is based on the vapour pressure data for the insecticides (Figure 8). The pyrethroid class of insecticides feature the lowest vapour pressures whilst the organophosphates, carbamates and DDT have the highest, second highest and third highest vapour pressure respectively. The vapour pressure data explains why the pyrethroids are generally less fugitive than the rest of the insecticides under oven ageing conditions conducted at 80°C. The low vapour pressure makes the pyrethroid group of insecticide the least volatile as compared to the rest of the insecticides and hence evaporation is slower than the rest. The present observations, however, suggest that pyrethroids are more stable towards thermal exposure compared with DDT and carbamates.

The chemical transformation of insecticides subjected to UV exposure is quite remarkable. Pyrethroids clearly exhibited significantly greater resilience to UV radiation than the rest of the insecticide classes. Thus the accelerated ageing tests conducted presently indicate that pyrethroids were a far more stable class of insecticides when exposed to typical environmental elements of degradation compared with the other classes of insecticides. This strongly suggests that high temperatures, ultra violet light and humidity are not the dominant mechanisms of oxidative/hydrolytic degradation of insecticides in the field.

**Figure 9:**
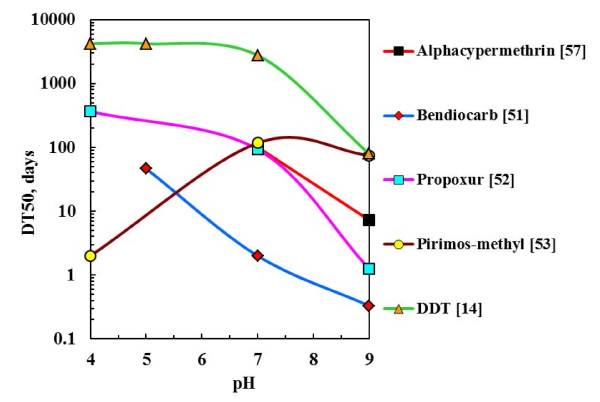
**Effect of pH on the rate of hydrolysis of WHO approved insecticides**. The rate of hydrolysis is expressed as half-life DT_50_, a measure of the time it takes for half the parent compound to transform.

It has been shown that, except for DDT, the other insecticides tested are very sensitive to pH-mediated hydrolysis. This is especially true at elevated pH values. The plots of the published half-life (DT50) values [14,51-53,57] against pH presented in Figure 9 show that the WHO-approved insecticides are more stable at lower pH values. The data presented in Figure 4 showed that some selected fillers had a stabilizing effect on alphacypermethrin dissolved in acetone containing 1 wt.% water. Similar results were obtained with bendiocarb although in this case greater stabilities were achieved. The stabilizing effect appeared to correlate with the surface pH of the mineral powders (indicated in brackets) suggesting they acted as acidic pH buffers. Phosphogypsum showed the best results. It is a cost-effective option and preliminary spray tests showed that it sticks well to a variety of vertical surfaces that are encountered in rural dwellings.

This suggests that pH-catalysed hydrolysis is a degradation mechanism that is able to deactivate all the other insecticide classes at a rate that is significantly faster that DDT. Malaria control authorities in South Africa prefer to spray DDT on mud walls commonly found in African rural rondavels whilst they spray pyrethroids on western style painted cement walls [61]. This preference is, in part, due to the fact DDT has the ability to persist longer on mud walls compared to pyrethroids. It is possible that in the field of application, surface chemical and biological properties of the walls being sprayed with the insecticides play a significant role in the breakdown of the insecticides through water mediated hydrolysis.

### Bioassays

Dwellings in deep rural areas commonly feature mud and dung coated walls. Bioassays were used to follow the time-dependent efficacy of the stabilized insecticides applied onto such surfaces under accelerated laboratory ageing conditions of high humidity and elevated temperature. The considerable variability in the data makes statistically reliable comparisons difficult. However, the results suggest that the carbamates bendiocarb and propoxur perform well when incorporated into an ordinary paint binder, while bendiocarb and alphacypermethrin precipitated on phosphogypsum also performed well in the accelerated ageing test conducted at 40°C and 90% relative humidity.

## Conclusions and recommendations

DDT has a significantly longer persistence than other WHO approved insecticides under IRS field conditions. Comparative tests were performed to track the stability of DDT and alternative insecticides under accelerated laboratory ageing conditions of elevated temperature, ultraviolet light exposure and high humidity. The results suggest that these factors are unlikely causes of the lower persistence of IRS insecticides in the environment. It is likely that water mediated hydrolysis, facilitated by a suitable environmental pH values and/or high bioactivity, are more important. To test this hypothesis, bio-assay experiments were conducted on mud and dung coated samples and aged at high humidity conditions that should favour rapid hydrolysis of the insecticides. It was found that selected insecticides incorporated into a conventional paint binder or adsorbed onto phosphogypsum provided effective life spans comparable to or exceeding that of DDT. Adsorbing an insecticide such as alphacypermethrin phosphogypsum mineral powder provided a suitable buffered pH environment, which minimized pH mediated hydrolysis. An acrylic paint binder stabilized carbamates but led to complete deactivation of the pyrethroids. The acrylic paint option provides for a translucent coating that may be more acceptable to users who do not want to alter the colour of their walls.

The results presented here relate to laboratory conditions. It will be necessary to conduct field trials in malaria invested areas to determine whether the present observations translate to actual field conditions. It is hoped that such trials will be able to shed light on the comparative performance of these formulations under normal field conditions. These findings may allow replacement of DDT, a persistent organic pollutant, with WHO-approved insecticides.

## Competing interests

There are neither any financial competing interests nor any non-financial competing interests (political, personal, religious, ideological, academic, intellectual, commercial or any other) to declare in relation to this manuscript.

## Authors' contributions

WWF contributed to the overall project supervision, planning of investigations and interpretation of results. MMS carried out the investigations and interpretation of results. FJWJL also contributed to the supervision of the project and the interpretation of results. NSN carried out the thermal analysis of insecticides and interpretation of the results. GWAB carried the gas chromatography analysis of insecticides aged in the presence of mineral powders. NASC carried out the statistical regression of bioassay results and interpretation of the results. MC supervised bioassay testing and interpretation of results. AM supervised the humidity ageing of the insecticide formulations. HFM and PHM contributed to the literature survey of the project and bioassay investigations. All authors read and approved this article.

## Author information

MMS is a postgraduate student studying MEng Chemical Engineering at the University of Pretoria. WWF is supervisor and project leader of this research. A full account of this research work in dissertation form can be found on http://upetd.up.ac.za/ETD-db/ETD-browse/browse.
